# A serum microRNA signature predicts trastuzumab benefit in HER2-positive metastatic breast cancer patients

**DOI:** 10.1038/s41467-018-03537-w

**Published:** 2018-04-24

**Authors:** Huiping Li, Jiang Liu, Jianing Chen, Huiyun Wang, Linbin Yang, Fei Chen, Siting Fan, Jing Wang, Bin Shao, Dong Yin, Musheng Zeng, Mengfeng Li, Jun Li, Fengxi Su, Qiang Liu, Herui Yao, Shicheng Su, Erwei Song

**Affiliations:** 1 Key Laboratory of Carcinogenesis and Translational Research (Ministry of Education), Department of Breast Oncology, Peking University Cancer Hospital and Institute, 52 Fucheng Road, Beijing, 100142 China; 2 Guangdong Provincial Key Laboratory of Malignant Tumor Epigenetics and Gene Regulation, Sun Yat-sen Memorial Hospital, Sun Yat-sen University, 107 Yanjiang West Road, Guangzhou, 510120 China; 3 Breast Tumor Center, Sun Yat-sen Memorial Hospital, Sun Yat-sen University, 107 Yanjiang West Road, Guangzhou, 510120 China; 4 State Key Laboratory of Oncology in Southern China, Tumor Center, Sun Yat-sen University, 651 Dongfeng East Road, Guangzhou, 510060 China; 5 Zhongshan School of Medicine, Sun Yat-sen University, 74 Zhongshan Road II,, 510080 Guangzhou, China

## Abstract

Trastuzumab is a standard treatment for HER2-positive (HER2^+^) breast cancer, but some patients are refractory to the therapy. MicroRNAs (miRNAs) have been used to predict therapeutic effects for various cancers, but whether miRNAs can serve as biomarkers for HER2^+^ metastatic breast cancer (MBC) patients remains unclear. Using miRNA microarray, we identify 13 differentially expressed miRNAs in the serum of HER2^+^ MBC patients with distinct response to trastuzumab, and four miRNAs are selected to construct a signature to predict survival using LASSO model. Further, our data show that miR-940 is mainly released from the tumor cells and miR-451a, miR-16-5p and miR-17-3p are mainly from the immune cells. All these four miRNAs directly target signaling molecules that play crucial roles in regulating trastuzumab resistance. In summary, we develop a serum-based miRNA signature that potentially predicts the therapeutic benefit of trastuzumab for HER2^+^ MBC patients and warrants future validation in prospective clinical trials.

## Abstract (web summary)

Resistance to therapy is a significant issue for patients with metastatic breast cancer (MBC). Here the authors analyze total miRNA from serum samples of 386 MBC patients before treatment with a follow up of 31 months and define a four miRNA signature that predicts the therapeutic benefit of trastuzumab.

## Introduction

Trastuzumab is a standard treatment for HER2-positive (HER2^+^) metastatic breast cancer (MBC) patients^1,2^. However, about 20–30% of patients undergoing first-line treatments are refractory to trastuzumab^3,4^, which is one of the most expensive oncology regimens that require prolonged administration. Therefore, there is a pressing need to identify the patients who benefit from trastuzumab before treatments. Several biomarkers, including HER2 amplicon, phosphoinositide-3 kinase signaling, estrogen receptor (ER) status, phosphatase and tensin homolog (PTEN) loss, insulin-like growth factor 1 receptor (IGF1R) signaling, and immune cell signatures, which are identified in primary breast tumors, have been associated with the trastuzumab sensitivity in early breast cancer patients undergoing neoadjuvant or adjuvant treatment^5–7^. However, in MBC patients, tumor samples are sometimes unavailable for biomarker examination. Even if biopsies are obtained, the accuracy and predictive power are greatly limited by tumor heterogeneity^8^. Hence, several serum predictive markers were studied in HER2^+^ MBC patients, including shed HER2 extracellular domain^9^, amphiregulin, epidermal growth factor, and transforming growth factor α^10^. Nevertheless, none of these parameters can predict trastuzumab response.

MicroRNAs (miRNAs) play crucial roles in regulating cancer biology. miRNAs in tumor samples can serve as biomarkers to predict the prognosis and treatment sensitivity in various tumor types^11–14^. Furthermore, emerging evidence suggests that the unique serum miRNAs can assist in early detection and diagnosis of malignancies^15,16^, as well as prognosis for cancer patient survival after surgery^17^. However, whether serum miRNAs can predict treatment response for targeted therapies in cancer patients remains unknown.

Here we assess associations between serum miRNA profiles and clinical outcome in serum samples from HER2^+^ MBC patients receiving first-line trastuzumab plus chemotherapy. Using LASSO Cox regression model, we define a signature that consists of four miRNAs. Patients with low-risk score are found to have better overall survival (OS) and progression-free survival (PFS) than those with high-risk score. The four serum miRNAs signature exhibits their predictive values in trastuzumab response and provides useful biomarkers for personalized therapy.

## Results

### Clinicopathological features of patients

Among the 386 patients with HER2^+^ MBC receiving first-line chemotherapy with trastuzumab, the median follow-up was 31 months (interquartile range [IQR] 19–40). The baseline clinicopathological features of patients in each cohort were well balanced in age, metastasis, molecular subtype, and treatment regimen, which are shown in Supplementary Table 1. The median follow-up of the 179 patients without trastuzumab was 24 months (IQR 16–35) and their baseline clinicopathological features did not differ from the patients receiving trastuzumab plus chemotherapy in this study (Supplementary Table 1). The treatment outcomes of the HER2^+^ MBC patients herein were comparable to the ones reported in previous studies^2,18–20^ (Supplementary Table 2). The study design is shown in Supplementary Figs. 1 and 2.

### Construction of a serum miRNA signature

**Fig. 1:**
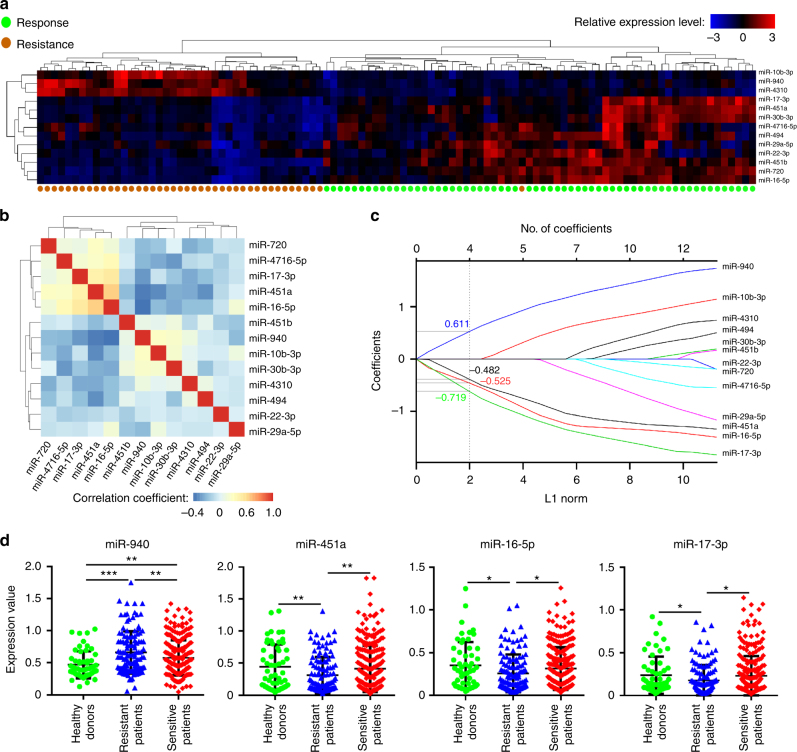
Construction of a four miRNA-based signature. **a** Hierarchical clustering of 13 serum miRNAs expression level in HER2^+^ MBC. Hierarchical clustering of serum samples from 61 patients responding to treatments (in green) and 42 patients resistant to treatments (in brown) with the 13 differentially expressed miRNAs using Euclidean distance and average linkage clustering. Each row represents an individual miRNA, and each column represents an individual sample. Pseudocolors indicate relative expression levels from low to high on a log2 scale from −3 to 3. *P*-values are *t*-tests on the log2-transformed values of the expression data. **b** Hierarchical clustering shows the collinearity of 13 candidate miRNAs. Correlation matrix heatmap of 13 miRNAs in the training cohort, where each cell represents the Pearson correlation between the row and column corresponding miRNAs. The legend shows the color change along with the change of correlation coefficient from −0.4 to 1.0. **c** LASSO coefficient profiles of the 13 HER2^+^ MBC-associated serum miRNAs. Each curve corresponds to a miRNA; the vertical line is drawn at the value *λ* = 0.11 chosen by 200-time cross-validation via 1-SE criteria. **d** The expression levels of the four miRNAs in serum of 55 healthy volunteer donors, 134 trastuzumab-resistant patients, and 252 trastuzumab-sensitive patients. Error bars in graphs are standard deviation (s.d). *P < 0.05; **P < 0.01; ***P < 0.001. *P*-values were obtained using two-tailed Student’s *t*-test

To identify the serum miRNAs that are associated with trastuzumab treatment response in an unbiased manner, we obtained data from 103 patients randomly selected from 254 patients of Sun Yat-sen Memorial Hospital as the training cohort, with 61 patients responding to the treatment and 42 patients resistant. We identified 13 differentially expressed miRNAs (*P* < 0.05) from all miRNA candidates detected by miRNA microarray. Among them, 3 miRNAs were downregulated (miR-10b-3p, miR-940, and miR-4310), and 10 (miR-17-3p, miR-451a, miR-30b-3p, miR-4716-5p, miR-494, miR-29a-5p, miR-22-3p, miR-451b, miR-720, and miR-16-5p) were upregulated in the sensitive patients (Fig. 1a).

In the training cohort, the expression of the 13 miRNAs was further evaluated by quantitative reverse transcriptase-PCR (qRT-PCR) analysis. However, we observed collinearity among some miRNAs (Fig. 1b), which would prejudice the results of traditional Cox regression analysis. Therefore, we used LASSO Cox regression model to select prognostic miRNAs to predict the PFS of the patients with 200 bootstrap replicates via penalized maximum likelihood (Supplementary Fig. 3), the regularization path was computed for the lasso at a grid of values for the regularization parameter lambda, which resulted in four (miR-451a, miR-16-5p, miR-17-3p, and miR-940) out of the 13 miRNAs and optimal weighting coefficients were selected to construct a prognostic signature (Fig. 1c). Based on the expression level of the four miRNAs, the following formula was derived to calculate disease progression risk score for each patient: Risk score = (0.611 × expression value of miR-940)−(0.482 × expression value of miR-451a)−(0.525 × expression value of miR-16-5p)−(0.719 × expression value of miR-17-3p).

To evaluate the serum levels of these 4 miRNAs in normal population, we collected serum samples from 55 healthy donors and assessed the expression levels of these 4 miRNAs. We found no statistical difference on the serum levels of miR-451a, miR-16-5p, and miR-17-3p between healthy individuals and HER2^+^ MBC patients sensitive to trastuzumab treatment. However, these 3 miRNAs decreased in the HER2^+^ MBC patients resistant to trastuzumab compared to the normal controls. In contrast, miR-940 increased in the HER2^+^ MBC patients sensitive to trastuzumab compared to healthy donors and further increased in the resistant patients (Fig. 1d).

### Evaluation of the risk score formula

**Fig. 2:**
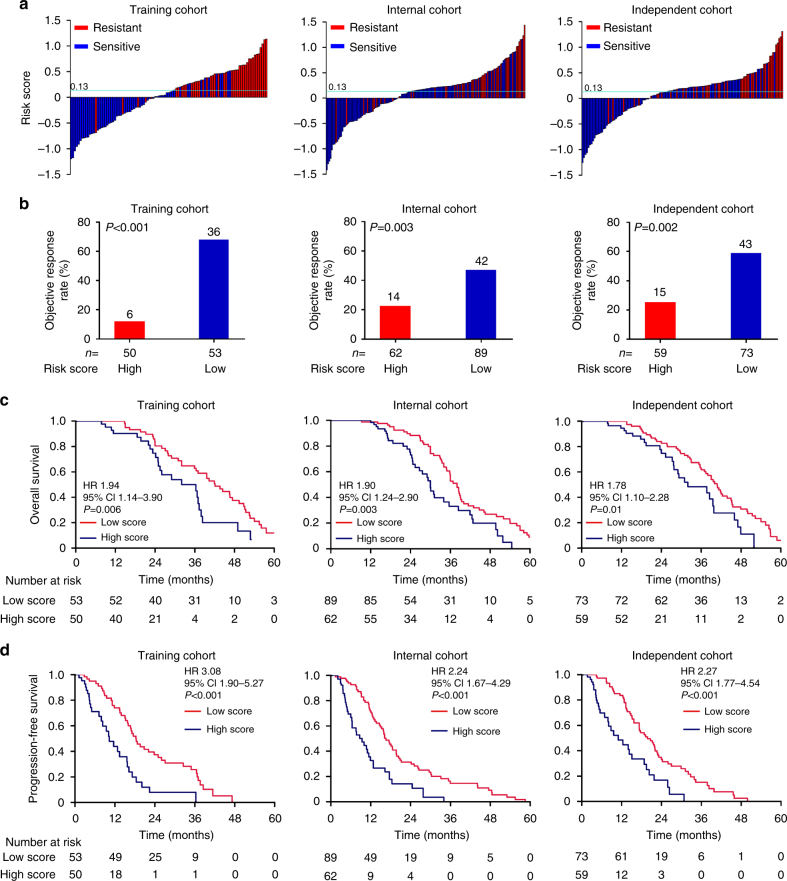
Distribution of the risk score and response status to trastuzumab treatment in three cohorts. **a** Waterfall plots for distribution of risk score and response status of individual patients. **b** Objective response rate between the high- and low-score groups. **c** Comparison of OS between high score and low score in the three cohorts. **d** Comparison of PFS between high score and low score in the three cohorts. We calculated hazard ratios (HR) and *P*-values with an univariate Cox regression analysis and the log-rank test, all statistical tests were two-sided. OS overall survival, PFS progression-free survival

With the risk score formula, patients in the training cohort were divided into high- and low-risk score subgroups according to the optimal selected cutoff score (0.13) determined by X-tile plots (Supplementary Fig. 4) based on association with PFS. Distribution of the clinicopathological characteristics did not vary significantly (*P* > 0.05) between the two subgroups (Table 1). When correlating the distribution of patient risk score with responding status, we found that the patients with low score generally had better response to trastuzumab-based treatment than those with high score (Fig. 2a). The objective response rate (ORR) was 12.00% (6/50) for the high-score subgroup, and 67.92% (36/53) for the low-score one (*P* < 0.001, Fig. 2b). Between patients with high and low score, the median OS was 31.8 vs 44.3 months (hazard ratio (HR) 1.94, 95% confidence interval (CI) 1.14–3.90; *P* = 0.006) (Fig. 2c), and the median PFS was 10.2 vs 18.9 months (HR 3.08, 95% CI 1.90–5.27; *P* < 0.001) (Fig. 2d).

**Table 1:** Clinical characteristics of patients in the cohorts from 386 HER2^+^ MBC patients receiving first-line chemotherapy with trastuzumab and 179 chemotherapy alone patients

	Chemotherapy/trastuzumab combination									Chemotherapy alone (*n* = 179)		
	Training (*n* = 103)			Internal (*n* = 151)			Independent (*n* = 132)					
	High score (*n* = 50)	Low score (*n* = 53)	*P*-value	High score (*n* = 62)	Low score (*n* = 89)	*P*-value	High score (*n* = 59)	Low score (*n* = 73)	*P*-value	High score (*n* = 67)	Low score (*n* = 112)	*P*-value
Median age	51	53	0.29	48	51	0.56	53	54	0.30	53	50	0.23
Range	37–74	35–79		28–75	31–78		31–73	29–78		31–85	28–78	
Menopause			0.40			0.92			0.61			0.45
Pre	19	15		22	31		13	20		21	28	
Post	31	38		40	58		46	53		46	84	
ECOG performance status			0.75			0.89			0.69			0.18
0	35	36		44	60		42	51		53	75	
1	13	16		16	26		15	21		12	34	
2	2	1		2	3		2	1		2	3	
Stage			0.91			0.88			0.57			0.72
I–II	24	27		28	37		30	34		29	55	
III	17	16		23	34		21	24		27	39	
IV	9	10		11	18		8	15		11	18	
ER status			0.16			0.61			0.49			0.74
Negative	18	27		21	36		27	29		33	51	
Positive	32	26		41	53		32	44		34	61	
PR status			0.68			0.13			0.57			0.95
Negative	19	23		20	40		28	31		30	52	
Positive	31	30		42	49		31	42		37	60	
Ki67 rate			0.83			0.98			0.72			0.74
≥15%	23	23		28	41		27	37		30	46	
<15%	21	25		30	42		28	33		33	59	
Unknown	6	5		4	6		4	3		4	7	
Metastatic sites			0.97			0.07			0.54			0.85
Soft tissue	37	41		49	45		43	39		41	69	
Visceral	40	41		47	53		44	60		47	66	
Bone	30	35		29	58		40	45		51	73	
CNS	3	4		4	3		5	4		7	8	
Number of metastatic sites			0.81			0.45			0.83			0.56
1	12	14		21	39		14	18		19	35	
2	19	19		24	28		23	30		25	47	
≥3	19	20		17	22		22	25		23	30	
Chemotherapy			0.99			0.85			0.98			0.65
Doxorubicin	11	16		11	20		13	18		18	26	
CTX	11	16		11	20		13	18		18	26	
Docetaxel	32	46		51	67		41	56		55	82	
Platinum	5	6		6	10		3	5		11	8	
Paclitaxel	7	11		9	8		11	13		10	24	
Capecitabine	10	13		16	17		9	17		15	21	
Vinorelbine	4	4		7	9		4	7		3	5	
Objective response			<0.001			<0.001			<0.001			0.59
CR	0	2		1	3		1	2		0	2	
PR	6	34		13	39		14	41		10	16	
SD ≥ 6 months	7	12		16	33		12	16		15	20	
SD < 6 months	5	1		10	3		6	5		17	23	
PD	32	4		22	11		26	9		25	51	

*P*-value from Chi-squared test or Fisher’s exact test for nominal categories

*ECOG* Eastern Cooperative Oncology Group, *ER* estrogen receptor, *PR* progesterone receptor, *CNS* central nervous system, *CTX* cyclophosphamide, *CR* complete response, *PR* partial response, *SD* stable disease, *PD* progressive disease

To further validate the performance of the four miRNA-based signature, we applied the formula and cutoff setting developed by qRT-PCR to the internal validation cohort with the remaining 151 out of the 254 patients. The clinicopathological characteristics were well balanced between the high- and low-score patients (Table 1). Similarly, in the internal cohort, patients with low score generally had better response to trastuzumab-based treatment than those with high score (Fig. 2a), and the ORR of the high-score patients was 22.58% (14/62) and 47.19% (42/89) for the low score group (*P* = 0.003, Fig. 2b). The median OS was 31.7 vs 37.7 months (HR 1.90, 95% CI 1.24–2.90; *P* = 0.003) (Fig. 2c), and the median PFS was 9.5 vs 17.8 months (HR 2.24, 95% CI 1.67–4.29; *P* < 0.001) (Fig. 2d).

We used a blinded external independent validation cohort to assess technical variability and the performance of the signature by using the same protocols and reagents. Similarly, in the independent validation cohort, patients with low score generally had better response to trastuzumab-based treatment than those with high score (Fig. 2a), and the ORR was 25.43% (15/59) for the high-score patients and 58.90% (43/73) for the low-score ones (*P* = 0.002, Fig. 2b). Also, between patients with high and low score, the median OS was 31.6 vs 41.7 months (HR 1.78, 95% CI 1.10–2.28; *P* = 0.01) (Fig. 2c), and the median PFS was 10.5 vs 21.3 months (HR 2.77, 95% CI 1.77–4.54; *P* < 0.001) (Fig. 2d).

### Univariate analysis and multivariate analysis in validation cohorts

**Fig. 3:**
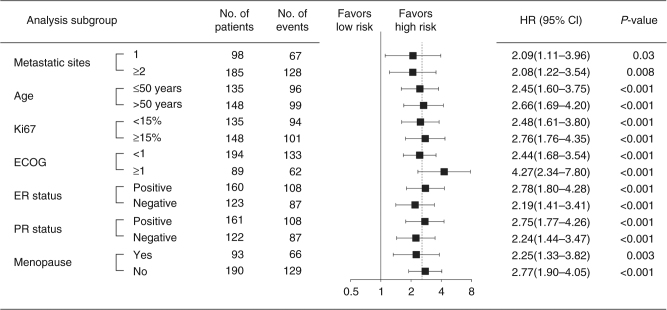
Forest plot for performance on PFS of predictive risk score stratified by clinicopathological features based on the Cox models. *P*-values were calculated using the two-sided log-rank test. HR and 95% CIs were given and visually represented by the squares and error bars. ECOG Eastern Cooperative Oncology Cohort, ER estrogen receptor, PR progesterone receptor, HR hazard ratio, 95% CI 95% confidence interval

Univariate analysis of the four single miRNA showed that each miRNA was independent prognostic factors in HER2^+^ MBC patients with trastuzumab treatment (Table 2). Furthermore, univariate and multivariate survival analysis were performed on the 4-miRNA signature and clinicopathological features for OS and PFS. Both the 4-miRNA signature and the metastatic sites were independent prognostic factors (Table 2). Upon stratified by the individual clinicopathological features, the signature was still a clinically and statistically significant applicable model in predicting PFS of the HER2^+^ MBC patients independent of the clinicopathological features, including the number of metastatic sites, age, Ki67, ECOG, ER status, PR status, and menopause (Fig. 3).

**Table 2:** Univariate and multivariate Cox regression analysis of 4-miRNA signature and clinicopathological characteristics with overall survival and progression-free survival in the combined two validation groups

	Univariate analysis		Multivariate analysis	
	HR (95% CI)	*P*-value	HR (95% CI)	*P*-value
Overall survival				
miR-940 (high vs low)	1.54 (1.25–2.23)	0.003	—	—
miR-451a (high vs low)	0.74 (0.38–0.96)	0.04	—	—
miR-16–5p (high vs low)	0.69 (0.36–0.90)	0.01	—	—
miR-17-3p (high vs low)	0.67 (0.32–0.87)	0.005	—	—
4-miRNA signature (high vs low score)	1.86 (1.35–2.55)	<0.001	1.88 (1.36–2.60)	<0.001
Metastatic sites (≥2 vs 1)	1.64 (1.37–1.98)	<0.001	1.65 (1.37–1.99)	<0.001
Age (>50 years vs ≤50 years)	1.06 (0.79–1.42)	0.69	0.82 (0.52–1.29)	0.39
Ki67(≥15% vs <15%)	1.26 (0.94–1.69)	0.12	1.10 (0.81–1.48)	0.54
ER (positive vs negative)	1.18 (0.88–1.58)	0.27	1.02 (0.70–1.49)	0.91
PR (positive vs negative)	1.25 (0.93–1.68)	0.15	1.13 (0.77–1.66)	0.53
Menopause (yes vs no)	1.19 (0.88–1.62)	0.26	1.36 (0.85–2.19)	0.20
Progression-free survival				
miR-940 (high vs low)	1.97 (1.38–3.13)	<0.001	—	—
miR-451a (high vs low)	0.71 (0.41–0.92)	0.02	—	—
miR-16-5p (high vs low)	0.64 (0.34–0.88)	0.003	—	—
miR-17-3p (high vs low)	0.59 (0.33–0.81)	0.001	—	—
4-miRNA signature (high vs low score)	2.58 (1.90–3.52)	<0.001	2.64 (1.92–3.65)	<0.001
Metastatic sites (≥2 vs 1)	1.67 (1.41–1.99)	<0.001	1.79 (1.48–2.15)	<0.001
Age (>50 years vs ≤50 years)	0.86 (0.65–1.14)	0.18	0.62 (0.40–1.02)	0.06
Ki67 (≥15% vs <15%)	1.14 (0.86–1.52)	0.35	0.92 (0.69–1.29)	0.61
ER (positive vs negative)	1.36 (1.03–1.80)	0.03	1.08 (0.74–1.56)	0.69
PR (positive vs negative)	1.25 (0.93–1.69)	0.13	1.09 (0.75–1.59)	0.65
Menopause (yes vs no)	1.09 (0.81–1.47)	0.56	1.67 (0.89–2.45)	0.07

*P*-values were calculated with the two-sided log-rank test

*HR* hazard ratio, *CI* confidence interval, *ER* estrogen receptor, *PR* progesterone receptor

### Comparison with other prognostic factors

**Fig. 4:**
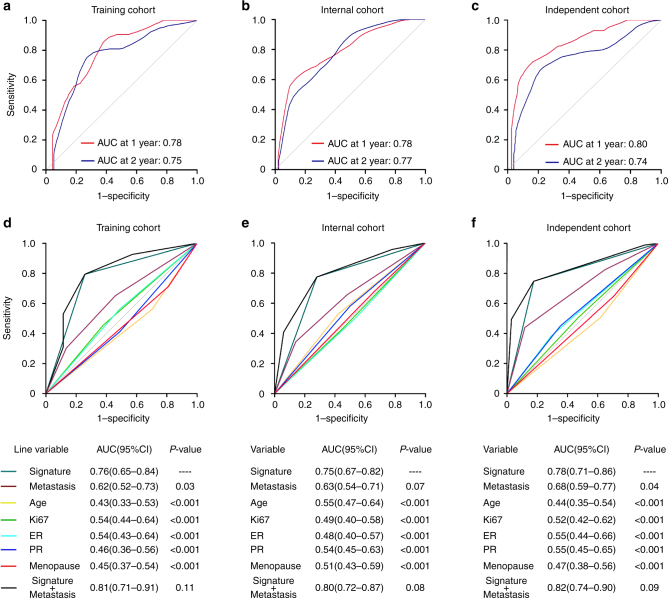
Receiver operating characteristic (ROC) curves and area under curve (AUC) values. **a**–**c** Time-dependent ROC curve in the training cohort, internal cohort, and independent cohort to assess prognostic accuracy; AUC at 1- and 2-year were calculated in the three cohorts according to the 4-miRNA signature, the spanning parameter of the NNE approach was span = 0.25×*n*_obs_^−0.20^. **d**–**f** Comparisons of the prognostic accuracy for trastuzumab response by the 4-miRNA signature (high score vs low score), metastatic sites (1, 2, ≥3), age (≤50 vs >50), Ki67 (>15% vs ≤15%), ER status (positive vs negative), PR status (positive vs negative), menopause (yes vs no), and 4-miRNA signature combining metastatic site. *P*-values showed the AUC of 4-miRNA signature vs the AUC of metastatic sites, age, Ki67, ER status, PR status, or menopause. AUC was calculated, and its 95% CI was estimated using Bootstrap method. The *P*-values were two-sided and based on Bootstrap test. ER estrogen receptor, PR progesterone receptor, HR hazard ratio, 95% CI 95% confidence interval

To further assess the accumulative effects of the 4-miRNA signature on risk score prediction, we calculated the area under the receiver operating characteristic curve (AUC) in the training, internal, and independent cohorts, respectively. Time-dependent receiver operating characteristic (ROC) analysis showed that the AUC for 1- and 2-year OS of 4-miRNA signature were 0.78 and 0.75 in training cohort (Fig. 4a), 0.78 and 0.77 in internal cohort (Fig. 4b), and 0.80 and 0.74 in independent cohort, respectively (Fig. 4c), suggesting that this signature is a stable predictor along with censored survival data. In ROC analysis, the AUC of risk score as categorical variable had no difference with continuous variable to predict trastuzumab response (Supplementary Fig. 5) and ruled out the concern of overfitted cutoff point. We compared the predictive accuracy of the 4-miRNA signature with each of the single miRNA, and the combined signature with the four miRNAs has higher AUC (0.74, 95% CI 0.69–0.81) and higher performance (specificity 73.16%, sensitivity 75.43%) as compared with any of the single miRNA or signature containing only three miRNAs (Supplementary Table 3). The signature was also significantly more specific and sensitive than other single clinicopathological risk factors (*P* < 0.05), except for the number of metastatic site in the internal cohort (*P* > 0.05) (Fig. 4d–f).

To develop a more sensitive predictive tool, we constructed a prognostic score model combining two independent prognostic factors, 4-miRNA signature and the number of metastatic sites, based on the training cohort. Combination of both of these factors had higher AUC than the 4-miRNA signature or metastatic site alone in all the thee cohorts, although the difference did not reach statistical significance (Fig. 4d–f).

### The predictive value of the 4-miRNA signature in trastuzumab response

**Fig. 5:**
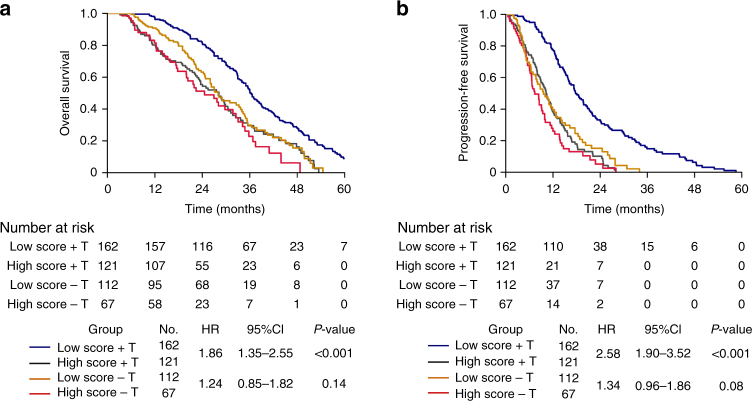
Kaplan–Meier survival analysis for patients with HER2^+^ MBC receiving only chemotherapy cohort and validation cohorts according to the 4-miRNA signature and trastuzumab treatment. **a** Overall survival. **b** Progression-free survival. *P*-values were calculated with the two-sided log-rank test. T trastuzumab

To explore whether the 4-miRNA signature is a specific predictive marker for the trastuzumab sensitivity or just a prognostic marker for a worse clinical outcome, we applied the formula and cutoff setting to stratified 179 HER2^+^ MBC patients receiving first-line chemotherapy without trastuzumab into the 4-miRNA signature high- and low-score groups. The clinicopathological characteristics were well balanced between the high- and low-score patients in this cohort (Table 1). We compared the clinical outcomes of the cohort receiving only chemotherapy with the validation cohorts (283) to evaluate whether this signature is predictive. The baseline clinicopathological features of patients in the cohort receiving only chemotherapy and the validation cohorts were well balanced in age, metastasis, molecular subtype, and treatment regimen, which are shown in Supplementary Table 1. As expected, the patients receiving trastuzumab plus chemotherapy had extended OS (HR 0.56; 95% CI 0.44–0.70; *P* < 0.001) and PFS (HR 0.45; 95% CI 0.36–0.56; *P* < 0.001) (Supplementary Fig. 6), compared to those receiving chemotherapy alone. In the subgroup analysis, low-score subgroup was associated with improved OS (HR 1.86; 95% CI 1.35–2.55; *P* < 0.001) and PFS (HR 2.58; 95% CI 1.90–3.52; *P* < 0.001) in patients receiving trastuzumab (Fig. 5). In contrast, OS and PFS had no significant difference between the high- and the low-score subgroup in patients receiving chemotherapy alone (Fig. 5 and Supplementary Table. 4). The *P*-values for interaction analysis between trastuzumab treatment status and the risk score was significant in OS and PFS (*P*_(trastuzumab × high-risk score)_ < 0.001, Supplementary Table. 5). Collectively, these data suggested that the 4-miRNA signature is a predictive marker for trastuzumab response in HER2^+^ MBC patients.

### Origin and mechanism of the four miRNAs

**Fig. 6:**
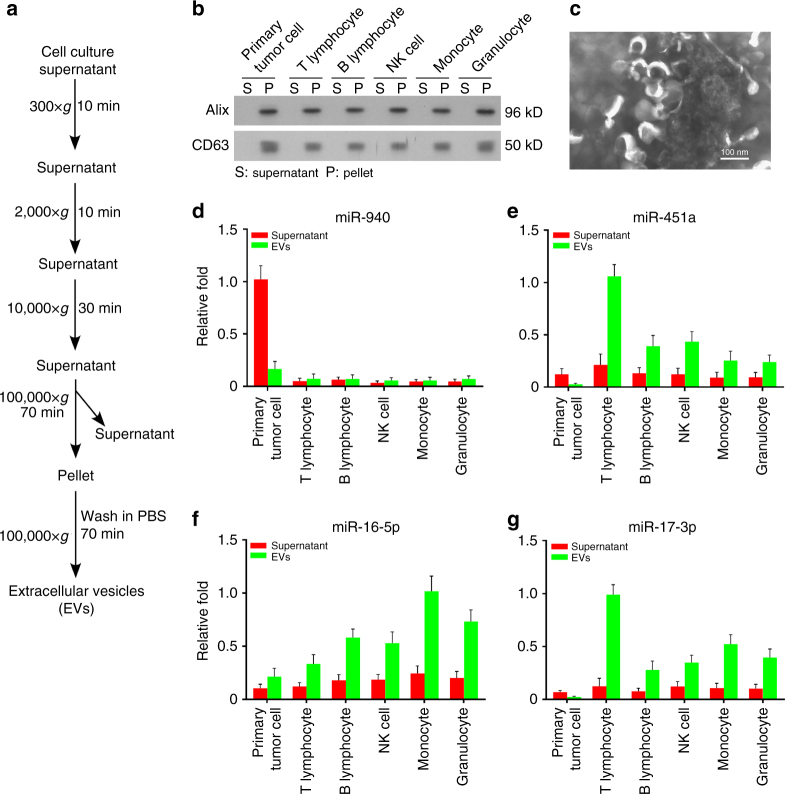
Origin of the biomarkers. **a** Strategy for the isolation of EVs and EV-free supernatants from culture supernatants based on differential ultracentrifugation. **b** Western blot analysis of CD63 and Alix in EVs prepared from pellets or supernatants. **c** Electron microscopy of purified EVs. Scale bar, 100 nm. **d**–**g** The relative expression levels of miRNAs in EVs and EV-free supernatants of cultured primary tumor cells, T lymphocytes, B lymphocytes, NK cells, monocytes, and granulocytes isolated from 5 patients (mean + s.e.m.)

To identify the origin of the four miRNAs, we isolated primary tumor cells from the surgical resected samples and autologous T lymphocytes, B lymphocytes, natural killer (NK) cells, monocytes, and granulocytes from the peripheral blood of 5 HER2^+^ primary breast cancer patients. We cultured the isolated tumor cells and immune cells at the density of 1 × 10^6^ cells ml^−1^ for 24 h. Thereafter, we isolated the miRNAs from extracellular vesicles (EVs) and EV-free supernatants in conditioned medium as previously reported (Fig. 6a)^21–23^. The isolated EVs were confirmed by EV markers CD63 and Alix (Fig. 6b) and transmission electron microscopy (Fig. 6c). Our data suggested that miR-940 expression was high in EV-free supernatants of the tumor cells. Whereas miR-451a and miR-17-3p were preferentially expressed in the EVs of T lymphocytes. In addition, high expression of miR-16-5p was observed in both the tumor cells and the hemopoietic components, wherein the EVs of monocytes exhibited the highest level of miR-16-5p (Fig. 6d–g). Collectively, our data suggested that miR-940 is mainly released from the tumor cells and miR-451a, miR-16-5p, and miR-17-3p are mainly from the EVs of immune cells.

**Fig. 7:**
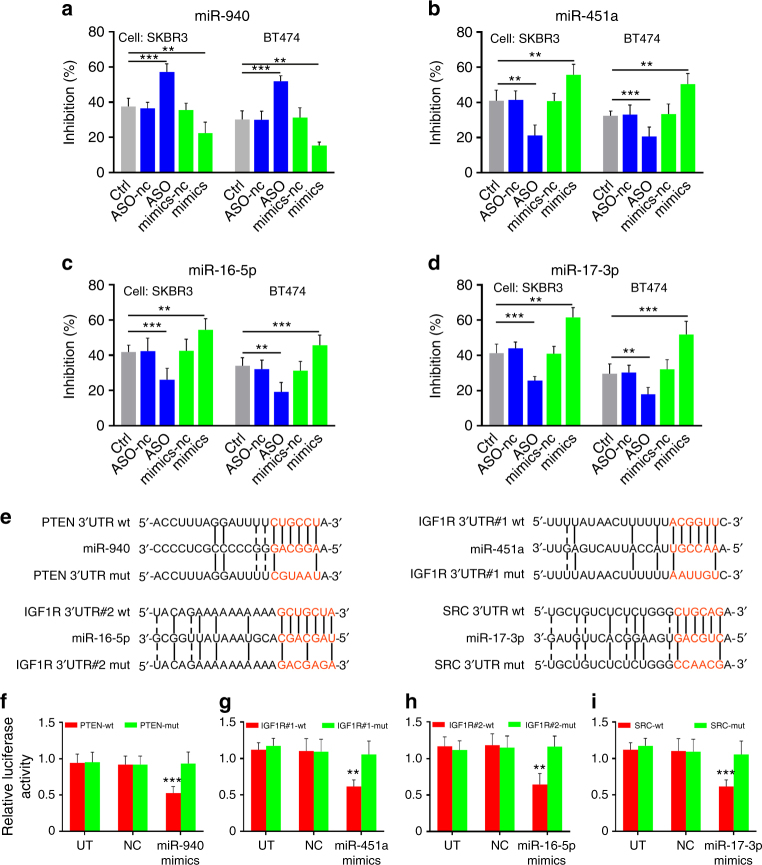
Mechanism of the biomarkers for trastuzumab treatment resistance. **a**–**d** SKBR3 and BT474 were transduced with nc, ASO, or mimics of four miRNAs, then trastuzumab was added to the medium at 10 μg ml^−1^ for 3 days, MTS assay was used to compare cell proliferation/apoptosis of treated cells (mean + s.e.m., *n* = 5 independent experiments; **P < 0.01; ***P < 0.001. *P*-values were obtained using two-tailed Student’s *t*-test). **e** TargetScan and MicroTar predicted PTEN as the target gene of miR-940, IGF1R as the target gene of miR-16-5p and miR-451a, and SRC as the target gene of miR-17-3p. Target sequences and their mutant forms of PTEN, IGF1R, and SRC in 3′UTR were synthesized and subcloned into pGL3 promoter vector. **f**–**i** Luciferase reporter assays for SKBR3 cells transfected with pGL3 promoter vectors carrying PTEN-3′UTR vs PTEN-mut-3′UTR, IGF1R-3′UTR vs IGF1R-mut-3′UTR or SRC-3′UTR vs SRC-mut-3′UTR in the absence or presence of the indicated miRNA mimics. (mean + s.e.m., *n* = 5 independent experiments; **P < 0.01; ***P < 0.001 compare to untreated cells by two-tailed Student’s *t*-test)

To investigate whether these four miRNAs contribute to trastuzumab sensitivity of HER2^+^ breast cancer cells, we transfected HER2^+^ breast cancer cells lines (SKBR3 and BT474) with corresponding miRNA mimics or miRNA anti-sense oligonucleotides (ASOs) using Lipofectamine 3000 and subsequently treated the cells with trastuzumab at 10 μg ml^−1^ for 3 days. Our data showed that transfection of tumor cells with ASOs against miR-940 or mimics of miR-451a, miR-16-5p, or miR-17-3p significantly increased cell death of tumor cells treated with trastuzumab compared to untreated tumor cells or those transfected with negative control oligonucleotides (nc). Consistently, overexpression of miR-940 or silencing miR-451a, miR-16-5p, or miR-17-3p markedly reduced the sensitivity of tumor cells to trastuzumab (Fig. 7a–d). Taken together, these data suggested that upregulation of miR-940 and downregulation of miR-451a, miR-16-5p, and miR-17-3p can induce trastuzumab resistance of tumor cells in vitro.

To investigate the potential targets of these miRNAs, we used target prediction tools TargetScan and MicroTar. Among the candidate target genes, PTEN was the potential target for miR-940, IGF1R for miR-451a and miR-16-5p, and SRC for miR-17-3p (Fig. 7e), as aberrant signaling of PTEN, IGF1R, and SRC has been involved in trastuzumab resistance of breast cancer cells^24–26^.

To further validate these targets, we generated reporter constructs containing the 3′ untranslated region (3′UTR) of these genes and co-transfected them with the corresponding miRNA mimics. Co-transfection with mimics of miR-940, miR-451a/miR-16-5p, and miR-17-3p specifically reduced luciferase activity of the SKBR3 cells transfected with reporter constructs containing PTEN-3′UTR, IGF1R-3′UTR, and SRC-3′UTR, respectively (Fig. 7f–i). Furthermore, mutations in the miRNA seed sequences completely abrogated luciferase reduction induced by the miRNA mimics, confirming the specificity of the miRNA target sites (Fig. 7f–i). Together, these data indicated that PTEN, IGF1R, and SRC are target genes of miR-940, miR-451a/miR-16-5p, and miR-17-3p, respectively.

## Discussion

Trastuzumab is a standard treatment for HER2^+^ MBC patients^27^. About 70% of these patients can benefit from first-line trastuzumab treatment, but most of them eventually develop resistance^3,4^. Given that HER2 is regarded as the only marker for anti-HER2-based treatment, there is a pressing need to identify patients who may respond to trastuzumab and to select the non-responders for alternative anti-HER2 regimens. In our present study, among three cohorts of HER2^+^ MBC patients receiving first-line trastuzumab-based therapy, we found that the expression of miR-940, miR-451a, miR-16-5p, and miR-17-3p constitutes a signature significantly associated with objective response rates, time to disease progression, and OS of the patients. With 600 μl blood samples from the patients, the 4-miRNA signature showed 73.16% specificity and 75.43% sensitivity to predict trastuzumab therapeutic effect. These findings highlight the potential utility of serum miRNA signature to predict therapeutic efficacy of trastuzumab for HER2^+^ MBC.

Our previous study and others have reported that a number of miRNAs are associated with trastuzumab resistance^28–30^. miR-21 expression in primary cancer tissue is associated with ineffective neoadjuvant trastuzumab therapy^29^. Moreover, an adjunct study evaluated circulating miR-21, miR-210, and miR-373 using qRT-PCR in the serum samples of 127 HER2^+^ breast cancer patients from Geparquinto trial before and after neoadjuvant therapy and 19 healthy controls and demonstrated changes in serum miR-21, miR-210, and miR-373 before and after treatment^30^. However, none of these studies were based on high-throughput screening in large-scale clinical samples in unbiased manner. Furthermore, the role of serum miRNAs in predicting trastuzumab response in HER2^+^ MBC patients has not yet been clarified.

Accumulating evidence suggests that the infiltrating lymphocytes and immune gene signatures in primary tumors predict trastuzumab benefit in HER2^+^ early^31,32^ and advanced breast cancers^33^. By analyzing EVs and EV-free supernatant from primary breast cancers cells and peripheral immune cells, we showed that miR-940 is mainly released from tumor cells and miR-451a, miR-16-5p, and miR-17-3p are mainly originated from EVs of immune cells. Consistently, miR-451a and miR-17-3p have been previously shown to be enriched in the EVs of T lymphocytes^34^ and miR-16-5p is overexpressed in peripheral blood mononuclear cells of prostate cancer^35^. More importantly, our data indicated that these four miRNAs were not only predictive biomarkers but also functional regulators of the sensitivity of breast cancer cells to trastuzumab. 3′UTR reporter assay showed that several signaling molecules that have been identified to play crucial roles in regulating trastuzumab resistance were the targets of these miRNAs. Taken together, our data suggested that the immune system plays a crucial role in trastuzumab efficacy^36–38^ and the EVs from circulating immune cells may also mediate trastuzumab sensitivity.

miR-16 was used as a endogenous control to evaluate circulating miRNA profile in various cancers, including breast cancer^39^. However, significant differences in serum miR-16 levels among different patients were also reported in ovarian cancer^40^, prostate cancer^41^, and melanoma^42^. This discrepancy may be due to different patient populations enrolled in different studies. It has been reported miR-16 was stable in a cohort of 83 breast cancer patients^39^. However, this study contained only six MBC patients and three HER2^+^ breast cancer patients. Moreover, blood cell contamination and hemolysis may impact miR-16 levels in serum^43^. Indeed, one of the biggest challenges in circulating miRNA study is the lack of reliable endogenous controls for all patients with different pathological contexts^44^. This is the reason why we used a spiked-in control miRNA in this study.

Trastuzumab is not available to every HER2^+^ MBC patients worldwide due to its high price. Therefore, our current study is limited because the inherent selection biases could be associated with the decision to administrate chemotherapy only or trastuzumab/chemotherapy combination, Furthermore, whether it is applicable to patients receiving trastuzumab and other anti-HER2 regimens (such as pertuzumab) and different ethnicities remains unclear. By accurate high-throughput screening of miRNAs in serum across a large cohort, we identify 4-miRNA signature as the first serum biomarker that can predict trastuzumab benefit in HER2^+^ MBC patients. Identification of additional biomarkers that increase its AUC is next research step to enhance its clinical utility. Furthermore, prospective studies in multicenter clinical trials are needed to further confirm the clinical validity and utility of the four miRNA signature as trastuzumab prediction biomarkers in the future.

Oncology isoforms of miRNAs, which can be detected by RNA-seq, are crucial in tumor progression. We designed the probes of microarray, which detected 1848 human miRNAs, based on the miRBase 18 released in 2011. The latest miRBase 21 released in 2014 contains 2588 human mature miRNAs, indicating fast and constant discoveries of new miRNAs. We used the microarray assay because it is of low cost, technically reliable, and practicable for clinical application in hospital. This is the major reason why most of studies with a large number of clinical samples use microarray technique to sequence the oncology miRNA profile^11,45,46^. Another advantage of microarray technique is that much less RNA input is required as compared to RNA-seq. This is very important in this scenario, as the miRNA expression in the serum is much less than the ones in tumors.

In summary, our data suggest that the serum-based 4-miRNA signature can effectively distinguish HER2^+^ MBC patients who are sensitive to trastuzumab from the resistant ones. As the techniques of examining serum miRNAs are now widely used in laboratory and clinics with reasonable cost of reagents, this signature may hold great promise for clinical application as biomarkers for HER2^+^ MBC patients to predict trastuzumab efficacy.

## Methods

### Patient enrollment

We collected serum samples from HER2^+^ MBC patients who met all the following criteria: (1) Histologically confirmed adenocarcinoma of the breast with metastatic disease. (2) HER2-positivity defined as 3(+) staining in immunohistochemistry (IHC) or amplification of fluorescence in situ hybridization (FISH, HER2/CEP17 ratio ≥2.0). (3) Ductal invasive breast cancer upon initial pathological diagnosis. (4) Received chemotherapy with or without trastuzumab as first-line treatments. (5) Life expectancy ≥3 months, performance status [on the Eastern Cooperative Oncology Cohort (ECOG) scale] ≤2. (6) All blood samples were collected from patients with informed consent, and the research program and all the related procedures were approved by Ethics committee of Sun Yat-sen Memorial Hospital, Sun Yat-sen University.

The serum samples of 386 patients treated with trastuzumab were collected before treatment from Sun Yat-sen Memorial Hospital (254 samples) for training and internal validation, and Peking University Cancer Hospital (54 samples) and Sun Yat-sen Tumor Center (78 samples) for blinded testing and external validation. A power calculation was performed for the training cohort using the assumptions: *α* of 0.05, *β* of 0.2, risk category population standard deviation of 0.8, an HR of 2.0 for the high-risk score cohort, and event probabilities of 0.5. The power calculation resulted in an estimated sample size of 103. We used random numbers to assign 103 out of the 254 patients from Sun Yat-sen Memorial Hospital to the training cohort, leaving the remaining population of patients from this center to the internal validation cohort. Additionally, serum samples from 179 patients who received chemotherapy without trastuzumab were collected from Sun Yat-sen Memorial Hospital (163 samples) and Peking University Cancer Hospital (16 samples). Serum samples from 55 healthy volunteer donors were collected from Sun Yat-sen Memorial Hospital for control. Trastuzumab was given based on 4 mg kg^−1^ loading dose and then 2 mg kg^−1^ weekly or 8 mg kg^−1^ loading dose and then 6 mg kg^−1^ every 3 weeks. The chemotherapy regimen was administered according to normal clinical practice based on ASCO guidelines. The response rate was evaluated using the Response Evaluation Criteria in Solid Tumors criteria version 1.1^47^. Treatment resistance was defined when diseases progressed in <6 months, while treatment response was determined when complete response and partial response or stable disease prolonged for >6 months. The above definitions adopted the criteria used in various previous clinical trials with or without trastuzumab treatment in MBC^2,20,48^. This study was approved by the ethic committee of each hospital and conducted in accordance with the REMARK criteria^49^. Informed consent for the use of blood sample for research purposes was obtained from patients and approved by Ethics committee of Sun Yat-sen Memorial Hospital, Sun Yat-sen University.

### Serum sample collection and processing

We followed Early Detection Research Network serum operating standard operating procedure strictly to collect and process serum samples: (1) Collected blood samples from patients at a fasting state into a serum separator tube, labeled the tube, and gently inverted the tube 8–10 times. (2) Allowed the blood to clot by leaving tube undisturbed at room temperature for 20 min. (3) Centrifuged the tube at 1500 × *g* for 10 min in a refrigerated centrifuge. (4) Transferred the liquid component (serum) into a RNase-free cryovial tube with transfer pipette and labeled the tube carefully. (5) Froze serum at −80°C for next step experiments, and the samples were maintained on ice while handling. To detect the hemolysis, we used the spectrophotometer to measure oxyhemoglobin absorbance at *k* = 414 nm. Hemolyzed serum samples that showed a peak at 414 nm would not be used for next step analysis.

### miRNA extraction

A fixed volume of serum (600 μl per sample) from each patient was used for miRNA profiling and quantitative real-time PCR detection. Total RNA was extracted with the Plasma/Serum Circulating and Exosomal RNA Purification Mini Kit (51000, NorgenBiotek Corporation, Canada), following the manufacturer’s instructions. Prior to RNA isolation from serum, a non-human miRNA, *cel*-miR-39, was spiked into the serum with a final concentration of 0.05 nM as a reference. At last, the extracted RNA dissolved in 100 μl nuclease-free water and eluted from spin-column was first dried by vacuum centrifugation at low temperature and then re-dissolved in RNA dephosphorylation reaction buffer for miRNA microarray analysis or nuclease-free water for qRT-PCR analysis.

### miRNA microarray

We used a non-commercial microarray assay as previously reported^11,45,50,51^. Briefly, the probes (40 μM final concentration) mixed with printing buffer were printed onto slides in duplicate using SmartArrayer™ 136 printer (CapitalBio Inc, Beijing, China); RNA extracted from 600 μl serum was dephosphorylated and labeled with 100 nmol l^−1^ of pCp-DY647 (Dharmacon, Lafayette, CO, USA) and 15 units of T4 RNA ligase (USB, Cleveland, Oh, USA) in a total reaction volume of 20 µl at 16 °C overnight. The labeled RNAs were mixed and hybridized to the array with a 2× hybridization solution (final concentration: 5× Denhart’s solution, 0.5% sodium dodecyl sulfate (SDS), 5× SSC) in a Hybridization Chamber (Corning Inc, Corning, NY, USA) at 46 °C for 12–16 h. After washing, slides were scanned with a LuxScan 10 K Microarray Scanner (Capital Bio, Beijing, China), and images were analyzed with th GenePix Pro 6.0 software (Axon Instruments, Foster City, CA, USA). Expression data were normalized through quantile normalization and the RMA algorithm. Then miRNAs with significantly different expression between sensitive and resistant patients were selected. The microarray data have been deposited in the Gene Expression Omnibus database (GSE101841).

### qRT-PCR analysis

RNA extracted from 600 μl serum of each sample was reversely transcribed with the Mir-X™ miRNA First-Strand Synthesis Kit (638313, Clontech Laboratories, USA). The RT reaction was combined in a 0.2 ml tube: 5 μl mRQ Buffer (2×), 3.75 μl RNA sample, and 1.25 μl mRQ enzyme. The tube was incubated in a thermocycler for 1 h at 37 °C, 5 min at 85 °C, then 90 μl ddH_2_O was added to bring the total volume to 100 μl. Subsequently, qRT-PCR with SYBR Green master mix (RR820A, TaKaRa, Japan) was performed with a LightCycler 480 thermocycler (Roche Diagnostics, Germany). The PCR reaction was combined in 384 plates: 5 μl SYBR Premix (2×), 0.4 μl miRNA-specific primer (10 μM, sequence is listed in Supplementary Table. 6), 0.4 μl mRQ 3′Primer (10 μM), 1 μl template, and 3.2 μl ddH_2_O. For inter-assay control, high (1 × 10^10^ copies per reaction), medium (1 × 10^8^ copies per reaction), and low (1 × 10^6^ copies per reaction) concentration of synthetic *cel*-miR-39 were used as template on each separate plate. Each sample was analyzed in 10 μl reaction volume in triplicate. PCR amplification consisted of an initial denaturation at 95 °C for 30 s, followed by 40 cycles of amplification at 95 °C for 5 s and 60 °C for 20 s.

### Primary tumor cell and immune cell isolation

Primary tumor cells from surgical resected samples and autologous T lymphocytes, B lymphocytes, NK cells, monocytes, and granulocytes from peripheral bloods of five HER2^+^ primary breast cancer patients were isolated as previously described^52–54^. Informed consent for the use of the samples was obtained from patients and approved by Ethics committee of Sun Yat-sen Memorial Hospital, Sun Yat-sen University. Briefly, whole blood was diluted with phosphate-buffered saline (PBS) and then centrifuged at 450 × *g* for 20 min above Ficoll. After centrifugation, peripheral blood mononuclear cells (PBMCs) were collected from the cell layer in the interface above Ficoll for lymphocyte and monocyte isolation, and the mixed granulocyte and erythrocyte suspension was under Ficoll. Monocytes, T lymphocytes, B lymphocytes, and NK cells were purified from PBMC fraction by CD14 MicroBeads (130-050-201, Miltenyi), CD3 MicroBeads (130-050-101, Miltenyi), CD19 MicroBeads (130-050-301, Miltenyi), and CD56 MicroBeads (130-050-401, Miltenyi), respectively, according to the manufacturer’s instructions. Erythrocytes in the mixture were lysed with ammonium chloride buffer for granulocyte isolation. Fresh primary breast tumor specimens of HER2^+^ breast cancer patients were taken directly from surgery for primary tumor cell isolation as previously described^55^. We cultured the isolated tumor cells and immune cells at the density of 1 × 10^6^ cells ml^−1^.

### **Cell culture and** in vitro **cell proliferation/apoptosis assay**

Two HER2^+^ cell lines SKBR3 and BT474 obtained from the American Type Culture Collection (ATCC) were used to investigate whether these four miRNAs contribute to trastuzumab sensitivity. Cells were maintained in RPMI 1640 with 10% fetal bovine serum (FBS). Transfection of the cells with miRNA mimics or miRNA ASOs (Genepharma, Supplementary Table. 7 and 8) was performed using Lipofectamine 3000 (Invitrogen) as we previously described^56^. Trastuzumab (Herceptin) was obtained from Genentech and dissolved in sterile water and added to medium at 10 μg ml^−1^ for 3 days. We performed cell proliferation/apoptosis assay using the CellTiter 96 AQueous Cell Proliferation Assay Kit (Promega) and calculated the percentage of inhibition of cell proliferation as [1−(treated cells/untreated cells)] × 100%. The cell lines were authenticated by short tandem repeat profiling and tested to exclude the mycoplasma contamination by PCR.

### Isolation and characterizations of EVs

In all, 1–3 × 10^6^ isolated primary breast tumor cells and immune cells were grown in culture medium supplemented with 10% FBS (endogenous EVs was depleted by overnight centrifugation at 100,000 × *g*) for 24 h. EVs from total culture supernatants were isolated by differential centrifugation as previously described^21,23^. Briefly, the supernatants were collected for centrifugation at 300 *g* for 10 min; 2000 × *g* for 10 min; 10,000 × *g* for 30 min; and 100,000 × *g* for 70 min. In all, 600 μl EV-free supernatant was prepared for RNA extraction, and the pellets were washed once with PBS and purified by centrifugation at 100,000 × *g* for 70 min. For RNA extraction, the final pellet containing EVs was re-suspended in 600 μl PBS and *cel*-miR-39 was spiked into EVs with a final concentration of 0.05 nM as a reference; RNA extraction and qRT-PCR analysis were performed as described above.

Characterizations of EVs were performed following the EV-TRACK guidelines^57^. For western blot analysis, the pellet was re-suspended in 30 μl RIPA lysis buffer and the protein yield was determined by the BCA assay (Thermo Scientific). Protein extracts were resolved through SDS-polyacrylamide gel electrophoresis, transferred to polyvinylidene difluoride membranes, and probed with antibodies against CD63 (Cat# sc-5275, Santa Cruz) and Alix (Cat# sc-53540, Santa Cruz). Peroxidase-conjugated anti-mouse antibody (CST) was used as secondary antibody and the antigen–antibody reaction was visualized by enhanced chemiluminescence assay (ECL, Thermo Scientific). For electron microscopy, EVs re-suspended in 10 µl PBS were loaded on formvar/carbon-coated EM grids (200 mesh), then adsorbed EVs were fixed in 2% paraformaldehyde for 5 min at room temperature. After fixation, the EVs were directly negatively stained with 2% uranyl acetate. Grids were examined using a JEM-1400 Transmission Electron Microscope (JEOL, Tokyo, Japan).

### MiRNA target prediction and luciferase assay

The miRNA target prediction tool TargetScan (http://www.targetscan.org/index.html) and MicroTar (http://tiger.dbs.nus.edu.sg/microtar/) were used to identify potential miRNA targets. Target sequences and their mutant forms of PTEN, IGF1R, and SRC in 3′UTR were synthesized as DNA oligonucleotides (Supplementary Table 9) and subcloned into pGL3 promoter vector (Promega). SKBR3 were transfected with 10 pmol miRNA mimics or nc and co-transfected with 0.2 mg per well wild-type or mutant 3′UTR-luc, and pRL-TK vectors (0.01 mg per well) were co-transfected as endogenous controls for luciferase activity. After the cells were transfected for 24 h, they were lysed, and luciferase activities were measured using a Dual-luciferase Assay Kit (Promega).

### Statistical analysis

Statistical analysis was performed with the R software (version 3.3.2). We used two-tailed Student’s *t*-test and significance analysis of microarray to analyze the differential expression of miRNA detected by microarray. We used chi-square test for categorical variables and chi-square or Fisher’s exact test for contingency tables. Package “glmnet” was used to perform LASSO Cox regression model to select optimal weighting coefficients via penalized maximum likelihood and build a prognostic signature. For survival analysis, OS was defined as the time from first treatment to death for any cause; PFS was defined as time from first treatment to first progression of breast cancer or death for breast cancer or due to treatment. Cox regression was used to determine prognostic performance. Kaplan–Meier method and log-rank test were used to detect potential prognostic factors. Interaction test between trastuzumab treatment status and risk score was performed as described previously^58–60^. AUC was employed to demonstrate the sensitivity and specificity of different variables by risk estimation and AUC at different cutoff time was used to measure the predictive accuracy. The “pROC” package was used to perform ROC curve and analyze AUC. The “survivalROC” package was used to perform the time-dependent ROC curve analysis. Bootstrap test was used to compare the significance for AUC. All statistical tests were two sided and *P*-values <0.05 were considered to be significant.

### Data availability

The microarray data have been deposited in the GEO database under the accession code (GSE101841). We declare that all the other data supporting the findings of this study are available within the article and its supplementary information files and from the corresponding author upon reasonable request.

## Electronic supplementary material

Supplementary Information(PDF 2069 kb)
