# Supplementary material for: A serum microRNA signature predicts trastuzumab benefit in HER2-positive metastatic breast cancer patients
Source: Nat Commun. 2018 Apr 24;9:1614. doi: 10.1038/s41467-018-03537-w (PMC5915573; doi:10.1038/s41467-018-03537-w)
Supplement: Supplementary file 1 — Supplementary Information(PDF 2069 kb) [file 41467_2018_3537_MOESM1_ESM.pdf]

SUPPLEMENTARY INFORMATION

A Serum MicroRNA Signature Predicts Trastuzumab Benefit in HER2 Positive Metastatic Breast Cancer Patients

Li et al.

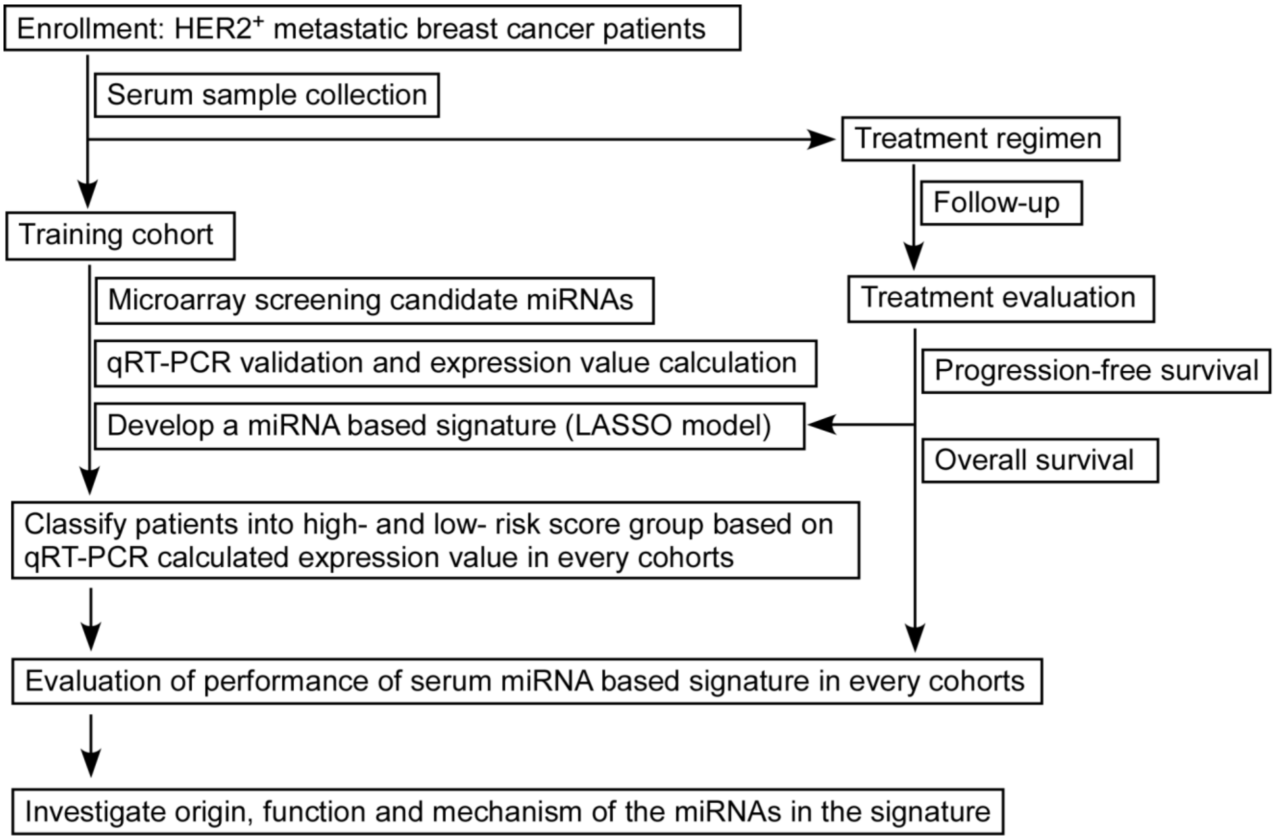

Supplementary Figure 1. Procedures and REMARK diagram for building predictive signature and validation in $HER2^+$ metastatic breast cancer patients.

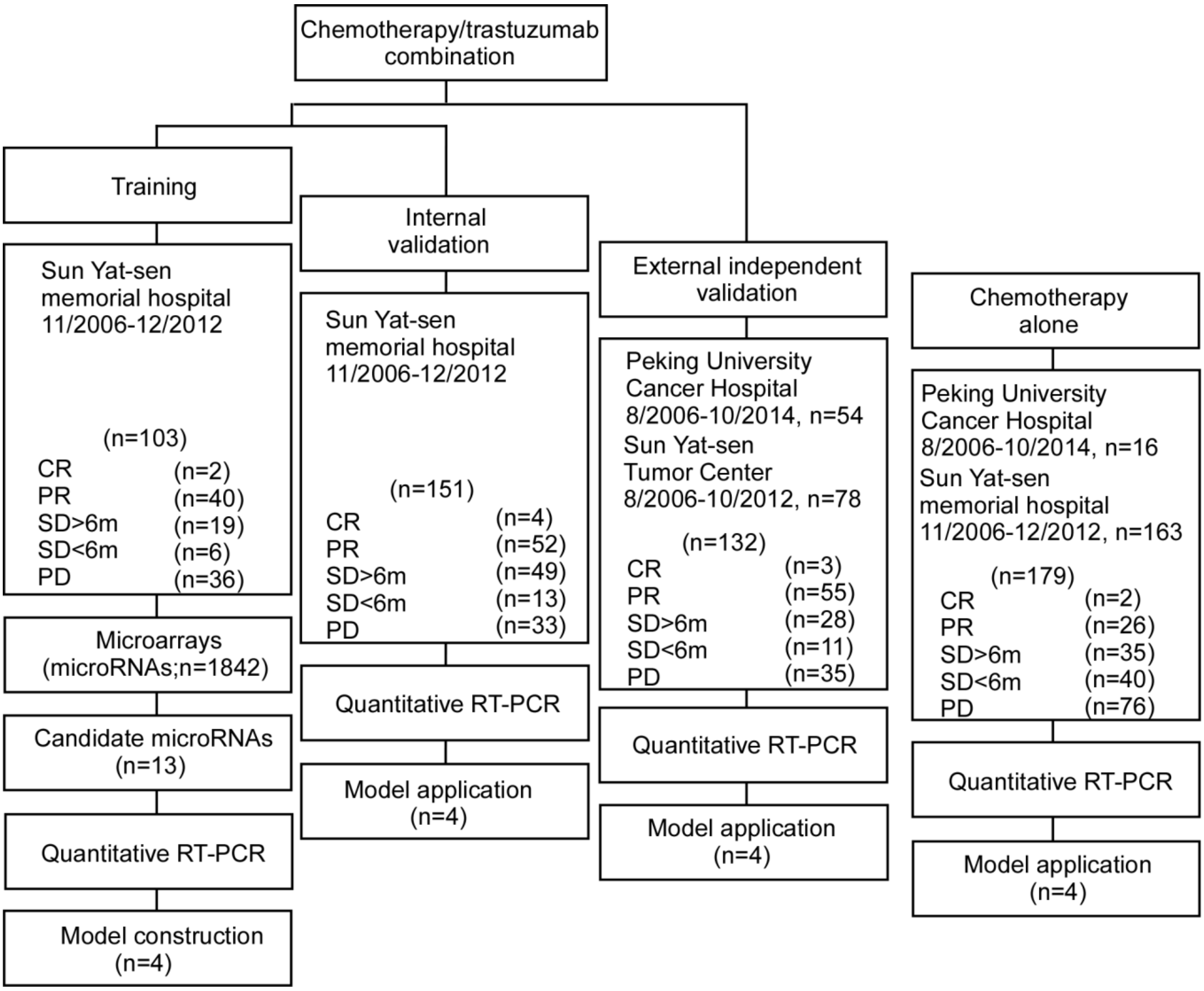

Supplementary Figure 2. Study design. Patients of CR, PR or SD prolonged more than 6 months were defined as sensitive patients. PD or SD prolonged less than 6 months were defined as resistant patients. CR = complete response, PR = partial response, SD = stable disease, PD = progressed diseases.

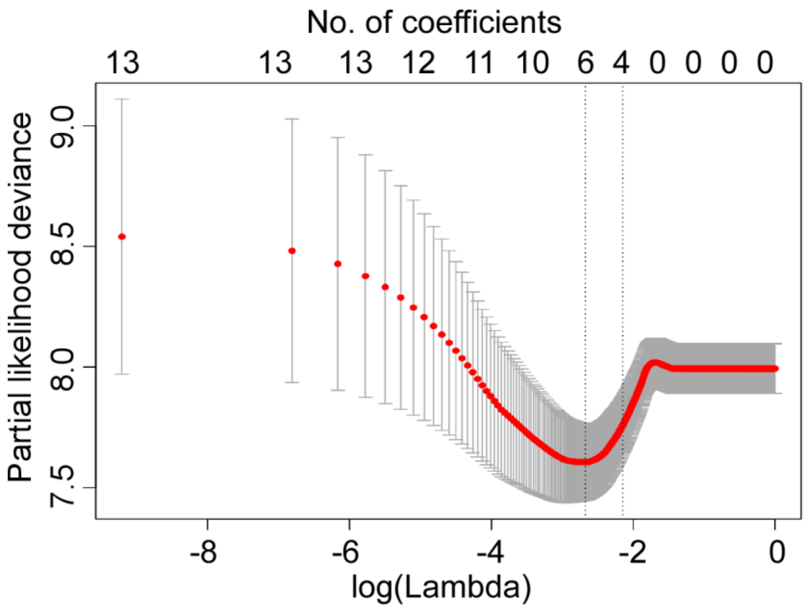

Supplementary Figure 3. Two hundred-time cross-validation for tuning parameter selection in the LASSO model corresponding to Fig. 1c. The solid vertical lines are partial likelihood deviance $\pm$ standard error, the two dotted vertical lines are drawn at the optimal values by minimum criteria (left) and 1-SE criteria (right). A value $\lambda = 0.11$ with $\log(\lambda) = -2.2$ is chosen by 200-time cross-validation via 1-SE criteria, and four coefficients are obtained at $\lambda = 0.11$, which gives the most regularized model such that error is within one standard error of the minimum.

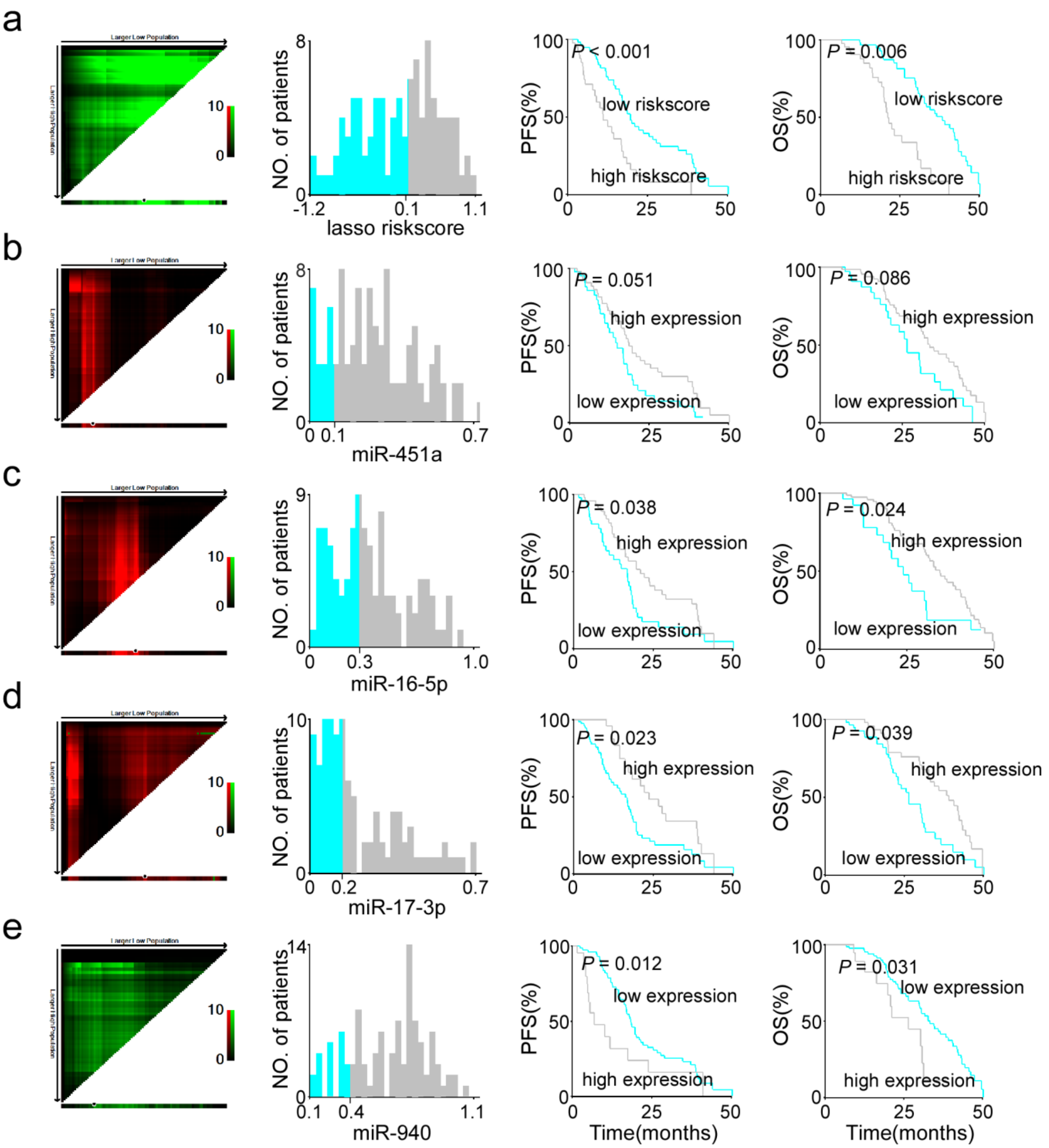

Supplementary Figure 4. X-tile plots of the four selected miRNAs and the LASSO risk value in the training cohort based on the association with the patients' PFS. Coloration of the plot (left panel) represents the strength of the association at each division, ranging from low (dark, black) to high (bright, red or green). Red represents inverse association between marker expression and survival, whereas green represents direct association. The values between green and gray in frequency histograms (second panel) were optimized cutoff value of high score and low score or high expression and low expression. Comparison of PFS (third panel) and OS (right panel) between four miRNAs-based signature high score and low score (**a**), or high expression and low expression of indicated miRNAs (**b-e**).

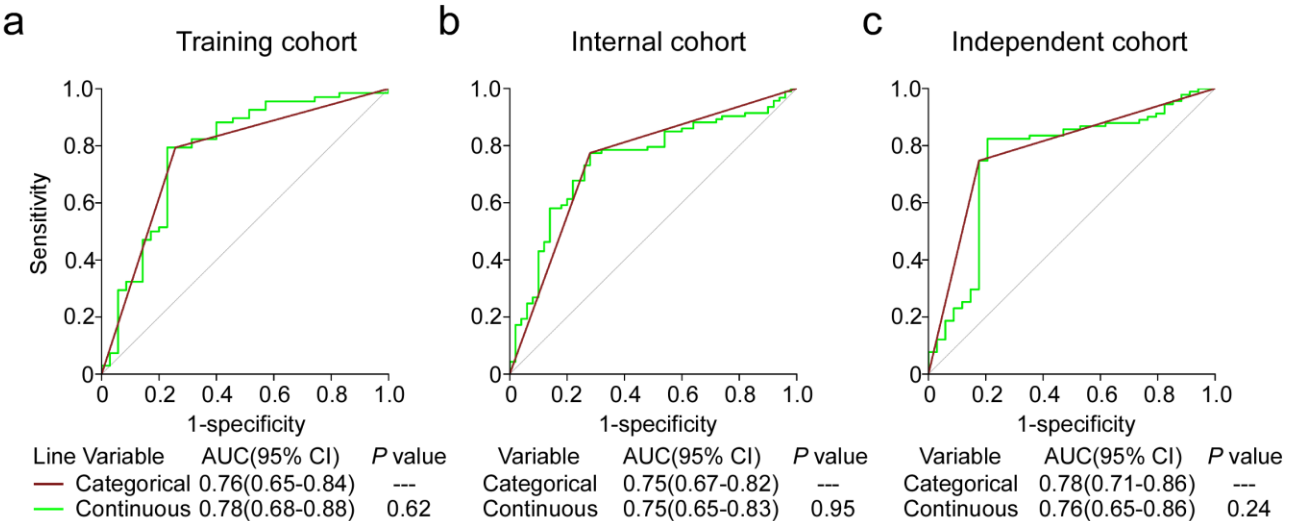

Supplementary Figure 5. Receiver operating characteristic (ROC) curves comparing performance of the risk score as a continuous variable with categorical variable in three cohorts. (**a**) Training cohort. (**b**) Internal cohort. (**c**) Independent cohort. AUC was calculated, and its 95% CI was estimated using Bootstrap method. The *P* values were two-sided and based on Bootstrap test. 95% CI, 95% confidence interval.

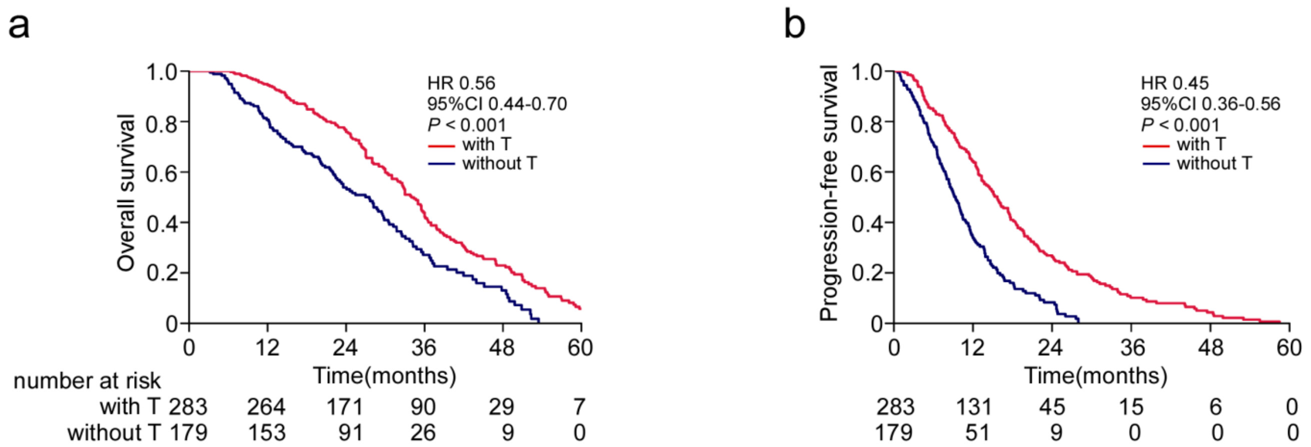

Supplementary Figure 6. Kaplan-Meier survival analysis for patients with HER2 positive metastatic breast cancer of the receiving only chemotherapy cohort (179) and the validation cohorts (283). (**a**) Overall survival. (**b**) Progression-free survival. We calculated hazard ratios and *P* values with Cox regression analysis and the two-sided log-rank test.

Alix

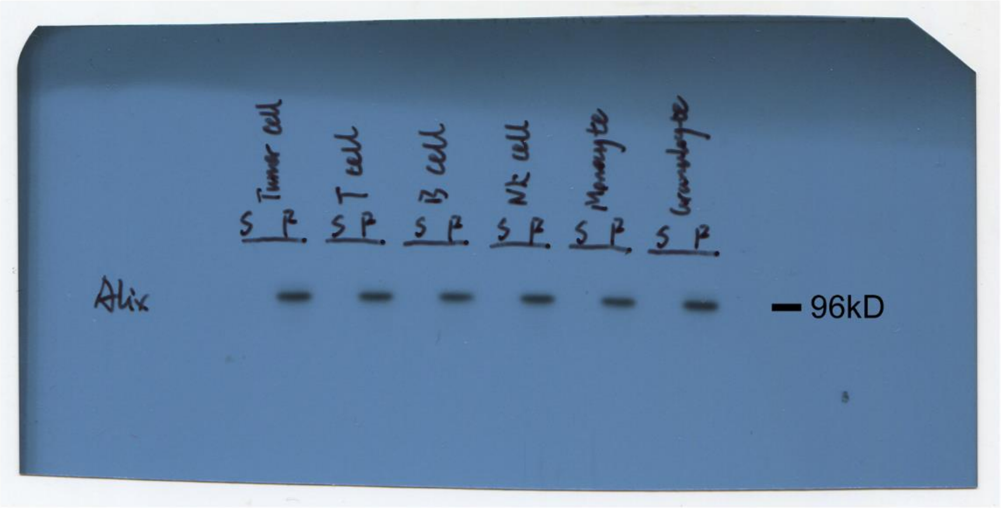

CD63

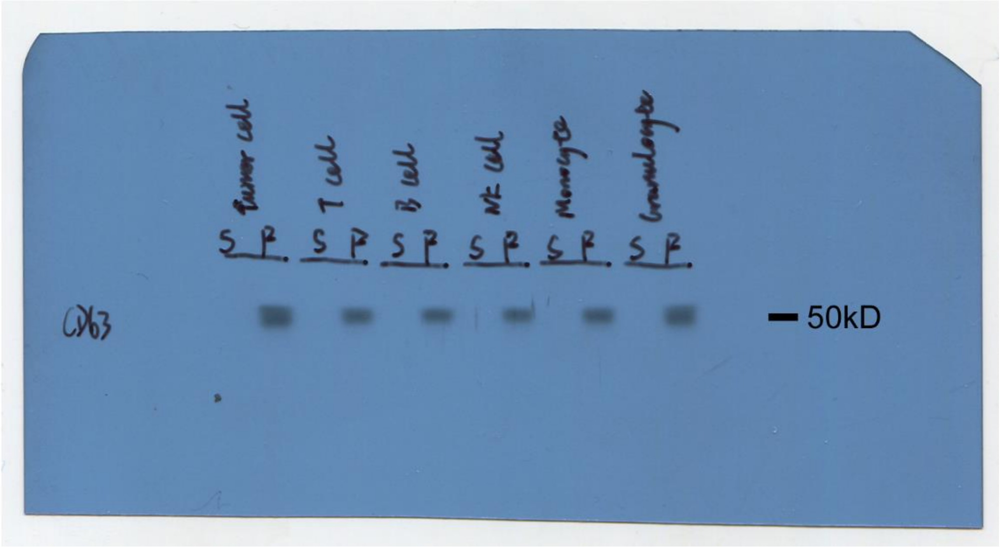

Supplementary Figure 7. Uncropped blots corresponding to Fig. 6b.

Supplementary Table 1. The clinical characteristics, treatment and response of patients in different cohorts.

	Chemotherapy/trastuzumab combination			Chemotherapy alone	*P* value
	**Training (n=103)**	**Internal (n=151)**	**Independent (n=132)**	**(n=179)**	
Median age , years	52(35-79)	50(28-78)	54(29-78)	51(28-85)	0.64
Period of $HER2^+$ MBC diagnosis					0.37
2006-2008	31(30.10%)	50(33.11%)	49(37.12%)	65(36.31%)	
2009-2011	59(57.28%)	81(53.64%)	58(43.94%)	83(46.37%)	
2012-2014	13(12.62%)	20(13.25%)	25(18.94%)	31(17.32%)	
Menopause					0.21
Pre-	34(33.00%)	53(35.10%)	33(25.00%)	49(27.37%)	
Post-	69(67.00%)	98(64.90%)	99(75.00%)	130(72.63%)	
ECOG performance status					0.98
0	71(68.93%)	104(68.87%)	93(70.46%)	128(71.51%)	
1	29(28.16%)	42(27.82%)	36(27.27%)	46(25.70%)	
2	3(2.91%)	5(3.31%)	3(2.27%)	5(2.79%)	
Stage					0.92
Ⅰ to Ⅱ	51(49.51%)	65(43.05%)	64(48.48%)	84(46.93%)	
Ⅲ	33(32.04%)	57(37.75%)	45(34.09%)	66(36.87%)	
Ⅳ	19(18.45%)	29(19.20%)	23(17.43%)	29(16.20%)	
ER status					0.41
Negative	45(43.69%)	57(37.75%)	56(42.42%)	84(46.93%)	
Positive	58(56.31%)	94(62.25%)	76(57.58%)	95(53.07%)	
PR status					0.66
Negative	42(40.78%)	60(39.74%)	59(44.70%)	82(46.37%)	
Positive	61(59.22%)	91(60.26%)	73(55.30%)	97(53.63%)	
Ki67 rate					0.65
≥15%	46(44.66%)	69(52.67%)	64(48.48%)	76(42.46%)	
<15%	46(44.66%)	72(47.68%)	61(46.21%)	92(51.40%)	
Unknown	11(10.68%)	10(6.62%)	7(5.31%)	11(6.14%)	
Metastatic sites					0.71
Soft tissue	78(75.73%)	94(62.25%)	82(62.12%)	110(61.45%)	
Visceral	81(78.64%)	100(66.23%)	104(78.79%)	113(63.13%)	
Bone	65(63.11%)	87(57.62%)	85(64.39%)	124(69.27%)	
CNS	7(6.80%)	7(4.64%)	9(6.82%)	15(8.38%)	
Number of metastatic sites					0.08
1	26(25.24%)	60(39.74%)	32(24.24%)	60(33.52%)	
2	38(36.89%)	52(34.44%)	53(40.15%)	62(34.64%)	
≥3	39(37.86%)	39(25.83%)	47(35.61%)	57(31.84%)	
Chemotherapy					0.86
Doxorubicin	27(26.21%)	31(20.53%)	31(23.48%)	44(24.58%)	
Cyclophosphamide	27(26.21%)	31(20.53%)	31(23.48%)	44(24.58%)	
Docetaxel	78(75.73%)	118(78.15%)	97(73.48%)	137(76.54%)	
Carboplatin/cisplatin	11(10.68%)	16(10.60%)	8(6.06%)	19(10.61%)	
Paclitaxel	18(17.48%)	17(11.26%)	24(18.18%)	34(18.99%)	
Capecitabine	23(22.33%)	33(21.85%)	26(19.70%)	36(20.11%)	
Vinorelbine	8(7.77%)	16(10.60%)	11(8.33%)	8(4.47%)	
Objective response					<0.001
Complete response	2(1.94%)	4(2.65%)	3(2.27%)	2(1.12%)	
Partial response	40(38.83%)	52(34.44%)	55(41.67%)	26(14.52%)	
Stable disease≥6 months	19(18.45%)	49(32.45%)	28(21.21%)	35(19.55%)	
Stable disease<6 months	6(5.83%)	13(8.60%)	11(8.33%)	40(22.35%)	
Progressive disease	36(34.95%)	33(21.85%)	35(26.52%)	76(42.46%)	

Abbreviations: ECOG, Eastern Cooperative Oncology Cohort; ER, estrogen receptor; PR, progesterone receptor; CNS, central nervous system

Supplementary Table 2. Comparison of median OS between our study and previous published studies.

Reports	Median OS(months)	
	**Chemotherapy alone**	**Chemotherapy + trastuzumab**
Our study	25(n=179)	34(n=386)
Baselga J, et al. *N Engl J Med*, 2012[1]	-----	32(n=406)
Dawood S, et al. *J Clin Oncol*, 2010[2]	21(n=118)	30(n=191)
Marty M, et al. *J Clin Oncol*, 2005[3]	22.7(n=94)	31.2(n=92)
Slamon D J, et al. *N Engl J Med*, 2001[4]	20.3(n=234)	25.1(n=235)

Supplementary Table 3. The performance of the 4-miRNA signature and indicated single miRNA in validation cohorts.

		Performance			***P* value**
		**AUC (95% CI)**	**Specificity(%)**	**Sensitivity(%)**	
4-miRNA signature		0.74(0.69-0.81)	73.16	75.43	-----
Single miRNA	miR-940	0.62(0.55-0.69)	54.63	68.42	0.003
	miR-451a	0.60(0.53-0.67)	47.42	70.17	< 0.001
	miR-16-5p	0.62(0.54-0.71)	72.31	51.19	0.003
	miR-17-3p	0.63(0.56-0.70)	60.89	67.64	0.004
Signature without	miR-940	0.66(0.57-0.75)	67.57	65.97	0.01
	miR-451a	0.69(0.62-0.76)	70.70	68.57	0.03
	miR-16-5p	0.67(0.58-0.75)	68.04	66.82	0.02
	miR-17-3p	0.66(0.58-0.74)	71.62	61.32	0.01

P values were two-sided and based on Bootstrap test. Abbreviations: AUC, area under the receive operating characteristic curve. 95% CI, 95% confidence interval.

Supplementary Table 4. Univariate and multivariate association analysis of 4-miRNA based signature, clinicopathological characteristics with overall survival and progression-free survival in the chemotherapy only group*.

	Univariate analysis		Multivariate analysis	
	**HR(95% CI)**	***P* value**	**HR(95% CI)**	***P* value**
**Overall survival**				
4-miRNA signature (High *vs* Low score)	1.24(0.85-1.82)	0.14	1.18(0.84-1.86)	0.33
Metastatic sites (≥2 *vs* 1)	1.71(1.35-2.16)	<0.001	1.69(1.34-2.14)	<0.001
Age (>50 years *vs* ≤50 years)	1.09(0.79-1.50)	0.59	1.04(0.66-1.63)	0.87
Ki67(≥15% *vs* <15%)	0.89(0.64-1.24)	0.50	0.87(0.61-1.23)	0.43
ER (Positive *vs* Negative)	1.02(0.74-1.40)	0.91	1.27(0.80-2.03)	0.32
PR (Positive *vs* Negative)	0.90(0.65-1.23)	0.50	0.77(0.48-1.24)	0.28
Menopause (Yes *vs* No)	1.07(0.75-1.53)	0.71	1.07(0.64-1.78)	0.81
**Progression-free survival**				
4-miRNA signature (High *vs* Low score)	1.34(0.96-1.86)	0.08	1.19(0.85-1.17)	0.30
Metastatic sites (≥2 *vs* 1)	2.02(1.56-2.61)	<0.001	2.07(1.59-2.69)	<0.001
Age (>50 years *vs* ≤50 years)	0.82(0.59-1.12)	0.22	0.66(0.43-1.02)	0.06
Ki67(≥15% *vs* <15%)	0.83(0.60-1.15)	0.26	0.71(0.50-1.00)	0.05
ER (Positive *vs* Negative)	1.02(0.74-1.41)	0.89	1.17(0.70-1.95)	0.56
PR (Positive *vs* Negative)	0.92(0.67-1.26)	0.59	0.86(0.51-1.45)	0.57
Menopause (Yes *vs* No)	0.93(0.65-1.33)	0.68	1.22(0.75-1.98)	0.43

*P values were calculated with the two-sided log-rank test. Abbreviations: HR, hazard ratio; 95% CI, confidence interval; ER, estrogen receptor; PR, progesterone receptor.

Supplementary Table 5. Interaction analysis between risk score subdivision and treatment modality on overall survival and progression-free survival in patients of two validation cohorts.

	Model 1		Model 2		Model 3		Model 4	
	HR (95% CI)	P value	HR (95% CI)	P value	HR (95% CI)	P value	HR (95% CI)	P value
**Overall survival**								
Trastuzumab *vs* chemotherapy	0.56(0.44-0.70)	<0.001			0.60(0.48-0.75)	<0.001	0.52(0.36-0.82)	<0.001
High score *vs* low score			1.62(1.30-2.07)	<0.001	1.59(1.27-1.98)	<0.001	1.19(0.82-1.72)	0.36
Trastuzumab×high score							1.61(1.39-2.09)	<0.001
**Progression-free survival**								
Trastuzumab *vs* chemotherapy	0.45(0.36-0.56)	<0.001			0.47(0.38-0.58)	<0.001	0.38(0.20-0.64)	<0.001
High score *vs* low score			2.11(1.72-2.60)	<0.001	2.00(1.63-2.47)	<0.001	1.35(0.97-1.88)	0.07
Trastuzumab×high score							1.94(1.47-2.74)	<0.001

P values were calculated with the two-sided log-rank test. Abbreviations: HR, hazard ratio; 95% CI, confidence interval.

Supplementary Table 6. Mature sequence of miRNAs and primer sequences for qPCR.

miRNA	Mature sequence	Primers for qPCR
cel-miR-39	5'-UCACCGGGUGUAAAUCAGCUUG-3'	5'-GTGCCGGTGTAAATCAGCTTG-3'
hsa-miR-17-3p	5'-ACUGCAGUGAAGGCACUUGUAG-3'	5'-CCCTGAAGGCACTTGTAGAAA-3'
hsa-miR-451a	5'-AAACCGUUACCAUUACUGAGUU-3'	5'-GCCGTTACCATTACTGAGTTAAA-3'
hsa-miR-4716-5p	5'-UCCAUGUUUCCUUCCCCCUUCU-3'	5'-TCCATGTTTCCTTCCCCCTTCT-3'
hsa-miR-940	5'-AAGGCAGGGCCCCCGCUCCCC-3'	5'-TATTTCCCCCGCTCCCCAAA-3'
hsa-miR-16-5p	5'-UAGCAGCACGUAAAUAUUGGCG-3'	5'-CGTTGGGTAAATATTGGCGAAA-3'
hsa-miR-22-3p	5'-AAGCUGCCAGUUGAAGAACUGU-3'	5'-GTCCCTGTTGAAGAACTGTAAA-3'
hsa-miR-720	5'-UCUCGCUGGGGCCUCCA-3'	5'-TCTCGCTGGGGCCTCCAAAA-3'
hsa-miR-451b	5'-UAGCAAGAGAACCAUUACCAUU-3'	5'-TAGCAAGAGAACCATTACCATT-3'
hsa-miR-30b-3p	5'-CUGGGAGGUGGAUGUUUACUUC-3'	5'-CTGGGAGGTGGATGTTTACT-3'
hsa-miR-10b-3p	5'-ACAGAUUCGAUUCUAGGGGAAU-3'	5'-GGGCGATTCTAGGGGAATAA-3'
hsa-miR-494	5'-UGAAACAUACACGGGAAACCUC-3'	5'-GAAACATACACGGGAAACCTC-3'
hsa-miR-29a-5p	5'-ACUGAUUUCUUUUGGUGUUCAG-3'	5'-GGATTTCTTTTGGTGTTCAGAA-3'
hsa-miR-4310	5'-GCAGCAUUCAUGUCCC-3'	5'-GCAGCATTCATGTCCCAAAAA-3'

Supplementary Table 7. Sequences of synthetic miRNA mimics oligo.

miRNA	Sense	Anti-sense
miR-940	5'-AAGGCAGGGCCCCCGCUCCCC-3'	5'-GGAGCGGGGGCCCUGCCUUUU-3'
miR-451a	5'-AAACCGUUACCAUUACUGAGUU-3'	5'-CUCAGUAAUGGUAACGGUUUUU-3'
miR-16-5p	5'-UAGCAGCACGUAAAUAUUGGCG-3'	5'-CCAAUAUUUACGUGCUGCUAUU-3'
miR-17-3p	5'-ACUGCAGUGAAGGCACUUGUAG-3'	5'-ACAAGUGCCUUCACUGCAGUUU-3'
mimics-nc	5'-UUCUCCGAACGUGUCACGUTT-3'	5'-ACGUGACACGUUCGGAGAATT-3'

Supplementary Table 8. Sequences of synthetic miRNA antisense oligo (ASO).

miRNA	ASO sequence
miR-940	5'-GGGGAGCGGGGGCCCUGCCUU-3'
miR-451a	5'-AACUCAGUAAUGGUAACGGUUU-3'
miR-16-5p	5'-CGCCAAUAUUUACGUGCUGCUA-3'
miR-17-3p	5'-CUACAAGUGCCUUCACUGCAGU-3'
inhibitor-nc	5'-CAGUACUUUUGUGUAGUACAA-3'

Supplementary Table 9. Synthesized DNA oligonucleotides of target sequences and their mutant forms.

	Synthesized DNA oligonucleotides
PTEN 3'UTR wild type	5'-ATGTACACCTTTAGGATTTT**CTGCCT**ACTCTATCCAGTTGTCCAAA-3'
PTEN 3'UTR mutation	5'-ATGTACACCTTTAGGATTTT**CGTAAT**ACTCTATCCAGTTGTCCAAA-3'
IGF1R 3'UTR#1 wild type	5'-GTCACTTTTATAACTTTTTT**ACGGTT**CAGATATTCATCTATACGTC-3'
IGF1R 3'UTR#1 mutation	5'-GTCACTTTTATAACTTTTTT**AATTGT**CAGATATTCATCTATACGTC-3'
IGF1R 3'UTR#2 wild type	5'-GTCTGTACAGAAAAAAAAAA**GCTGCTA**TTTTTTTTGTTCTTGATCTT-3'
IGF1R 3'UTR#2 mutation	5'-GTCTGTACAGAAAAAAAAAA**GACGAGA**TTTTTTTTGTTCTTGATCTT-3'
SRC 3'UTR wild type	5'-GCCCCTGCTGTCTCTCTGGG**CTGCAG**AGTCTGCCCCACATGTGGCCA-3'
SRC 3'UTR mutation	5'-GCCCCTGCTGTCTCTCTGGG**CCAACG**AGTCTGCCCCACATGTGGCCA-3'

Supplementary References

1. Baselga J, *et al.* Pertuzumab plus trastuzumab plus docetaxel for metastatic breast cancer. *The New England journal of medicine* **366**, 109-119 (2012).
2. Dawood S, Broglio K, Buzdar AU, Hortobagyi GN, Giordano SH. Prognosis of women with metastatic breast cancer by HER2 status and trastuzumab treatment: an institutional-based review. *Journal of clinical oncology : official journal of the American Society of Clinical Oncology* **28**, 92-98 (2010).
3. Marty M, *et al.* Randomized phase II trial of the efficacy and safety of trastuzumab combined with docetaxel in patients with human epidermal growth factor receptor 2-positive metastatic breast cancer administered as first-line treatment: the M77001 study group. *Journal of clinical oncology : official journal of the American Society of Clinical Oncology* **23**, 4265-4274 (2005).
4. Slamon DJ, *et al.* Use of chemotherapy plus a monoclonal antibody against HER2 for metastatic breast cancer that overexpresses HER2. *The New England journal of medicine* **344**, 783-792 (2001).
